# Seroprevalence of Chikungunya Virus, Jamaica, and New Tools for Surveillance

**DOI:** 10.3201/eid2808.220558

**Published:** 2022-08

**Authors:** Andre R.R. Freitas, Laura Pezzi, Luciano P.G. Cavalcanti, Fabrice Simon

**Affiliations:** Faculdade São Leopoldo Mandic, Campinas, São Paulo, Brazil (A.R.R. Freitas);; Unité des Virus Émergents, INSERM-IRD, Aix-Marseille Université, Marseille, France (L. Pezzi, F. Simon);; Universidade Federal do Ceará, Fortaleza, Brazil (L.P.G. Cavalcanti)

**To the Editor:** We read with great interest the recent article by Anzinger et al. (*1*), who found a seroprevalence of 83.6% for chikungunya in pregnant women in the metropolitan region of Kingston, Jamaica. These data are similar to the seroprevalence found nationwide by the Jamaica Health and Lifestyle Survey III, 2016–2017 (Ministry of Health and Welfare, Jamaica), which was 82% among women, 78.5% among men, and 80.4% overall. These values enable estimating a total of 2,187,325 chikungunya infections in Jamaica during the 2014 epidemic. The government of Jamaica reported 1,420 cases of chikungunya to PAHO in 2014 and no deaths (*2*), even correcting for the proportion of unapparent infections, the proportion of cases captured by passive surveillance was <0.1%. Although there were no officially reported deaths in Jamaica, 2 cases of newborn deaths from chikungunya were reported (*3*), and 1 study found 2,499 excess deaths (*2*) during the epidemic period. The increase in mortality was greater for the extremes of age, but it occurred in several age groups (*2*).

Anzinger et al.’s results reinforce the findings of Sharp et al. (*4*), who showed the importance of active surveillance to assess chikungunya burden. Through active surveillance implemented in Puerto Rico, it was possible to verify that 8% of symptomatic cases of chikungunya identified were captured by passive surveillance. In addition, passive surveillance identified 7 deaths, whereas active surveillance was able to confirm 31 deaths from chikungunya. However, 1,310 excess deaths were reported during the Puerto Rico epidemic in 2014 (*5*).

The introduction of chikungunya in the Americas has brought greater complexity to surveillance in the region, which includes some low-resource countries. It is essential to establish active and viable surveillance tools and, perhaps, new case definitions in order to better assess the population burden of this disease and the complications of acute and chronic cases.
